# The socio-economic burden of human African trypanosomiasis and the coping strategies of households in the South Western Kenya foci

**DOI:** 10.1371/journal.pntd.0006002

**Published:** 2017-10-26

**Authors:** Salome A. Bukachi, Simiyu Wandibba, Isaac K. Nyamongo

**Affiliations:** Institute of Anthropology, Gender and African Studies, University of Nairobi, Nairobi, Kenya; Common Heritage Foundation, NIGERIA

## Abstract

**Introduction:**

Human African Trypanosomiasis (HAT), a disease caused by protozoan parasites transmitted by tsetse flies, is an important neglected tropical disease endemic in remote regions of sub-Saharan Africa. Although the determination of the burden of HAT has been based on incidence, mortality and morbidity rates, the true burden of HAT goes beyond these metrics. This study sought to establish the socio-economic burden that households with HAT faced and the coping strategies they employed to deal with the increased burden.

**Materials and methods:**

A mixed methods approach was used and data were obtained through: review of hospital records; structured interviews (152); key informant interviews (11); case narratives (12) and focus group discussions (15) with participants drawn from sleeping sickness patients in the south western HAT foci in Kenya. Quantitative data were analysed using descriptive statistics while qualitative data was analysed based on emerging themes.

**Results:**

Socio-economic impacts included, disruption of daily activities, food insecurity, neglect of homestead, poor academic performance/school drop-outs and death. Delayed diagnosis of HAT caused 93% of the affected households to experience an increase in financial expenditure (ranging from US$ 60–170) in seeking treatment. Out of these, 81.5% experienced difficulties in raising money for treatment resorting to various ways of raising it. The coping strategies employed to deal with the increased financial expenditure included: sale of agricultural produce (64%); seeking assistance from family and friends (54%); sale/lease of family assets (22%); seeking credit (22%) and use of personal savings (17%).

**Conclusion and recommendation:**

Coping strategies outlined in this study impacted negatively on the affected households leading to further food insecurity and impoverishment. Calculation of the true burden of disease needs to go beyond incidence, mortality and morbidity rates to capture socio-economic variables entailed in seeking treatment and coping strategies of HAT affected households.

## Author summary

Sleeping sickness affects people often living in remote rural areas and those who mainly depend on subsistence agriculture. We carried out a study among former sleeping sickness patients in Kenya to find out the socio-economic challenges they faced in seeking treatment and the coping strategies they used to deal with them. This is important because the socio-economic effects of sleeping sickness and its coping strategies have not been adequately researched on yet it is on the strength of these impacts that policies and control programmes are formulated. If the real burden of sleeping sickness is not known, then it will continue to be neglected in terms of the attention it receives world-wide. Sleeping sickness patients and their households spent a lot of money seeking treatment besides facing challenges of disruption of daily activities, food insecurity, neglect of homesteads, poor academic performance/school drop-outs and death. Majority of them faced difficulties in raising the money required for seeking treatment hence resorted to various coping strategies. These negatively impacted on them and their households, already living on less than a dollar per day. There is need to pay attention to these effects of sleeping sickness in establishing the real burden of the disease.

## Introduction

Human African trypanosomiasis (HAT) also known as sleeping sickness is one of the 17 neglected tropical diseases identified by the World Health Organization (WHO) and has also been marked for elimination by the year 2020 [1,2]. Two forms of the disease exist depending on the parasite involved; *trypanosome brucei rhodesiense* that is dominantly found in the Southern and Eastern Africa regions, Kenya included and *Trypanosoma brucei gambiense* mostly found in Central and Western Africa [3]. It affects the world’s poorest and tends to occur in areas where there are no doctors, no drugs, hunger is greatest and food security least, incomes are lowest, health information is scanty and human need is greatest [4]. In 2002, calculations showed that HAT was the cause of loss of about 1.5 million DALYs, making it to be ranked number 7 in relation to other diseases [5]. It is ranked second among the top five priority zoonoses in Kenya [6]. The African region accounts for close to 90% of all the patients reported [7]. In spite of its significant presence in East and Central African region, there is a dearth of knowledge on the social and economic burden of African typanosomiasis. FAO estimates that Africa loses up to US$1.5 billion annually as a result of the disease [3]. African trypanosomiasis reduces labour resources, prevents growth of the livestock industry given that high yielding animals are less likely to survive the disease, affects availability of meat and milk and deprives farmers of draught power [8].

The disease generally occurs in remote rural areas where health systems are weak or non-existent and tends to affect economically active people [9]. The resulting burden on the extended family is heavy, not only because infected individuals become unproductive but also because close relatives have to spend time taking them for treatment and looking after them. Time and money spent on seeking treatment may be a serious drain on the family’s resources [10, 11]. Left untreated, the final outcome of the disease for the patient is death, but equally devastating is its effect on households, communities and quality of life resulting from its insidious and debilitating nature [12]. Economically, the effects of the disease are costly for young and developing economies like Kenya, which are predominately dependent on agriculture [13].

Studies in Uganda and Democratic Republic of Congo have demonstrated that HAT can have an adverse impact on the functioning of households [12, 14]. Such adverse consequences include: increased poverty; decline in agricultural activities often leading to famine or lack of basic food security; disruption of children’s education and; generally reversal of role obligations, which more often than not enhance women’s and children’s burdens [10, 15]. The disability adjusted life years (DALYs) has over the years been used to quantify the impact of disease. However, DALY calculations have been criticized for failing to capture the true burden of NTDS [16–19]. Studies conducted to estimate the disability adjusted life years (DALYs) of NTDs have focused more on morbidity and mortality rates [5, 20]. Given that the socio-economic effects of HAT and its coping strategies on households, have not been adequately researched in Kenya, yet it is on the strength of these impacts that policies and control programmes are formulated, this paper documents peoples' experiences to highlight *rhodesiense* HAT’s direct and indirect effects on affected households and how the coping strategies put in place by households further impoverishes them.

## Methods

### Ethics statement

Ethical clearance for this study was obtained from the WHO Ethical committee (M8/181/4/B.317) and from the Kenya Medical Research Ethical review board (KEMRI/RES/7/3/1). Ethical considerations were upheld throughout the study including obtaining written/thumb print consent from the respondents before the interviews.

### Description of the study site

The study was carried out in south western Kenya, an area that has been a foci for HAT experiencing epidemics since the late 1980’s. Most HAT cases prior to 1990 were from Lambwe valley in Nyanza province. However, during 1990’s-2002 the majority of cases came from new focus in Teso and Bungoma districts in south western Kenya [21]. The key villages in the study area that recorded high HAT numbers were: Alkudiet, Amongura, Amaase, Amoni, Amukura, Apatit, Bukhwamba, Ikapolok, Katelenyang, Kodedema, Kokoki, Obekai and Obuchun [21]. These were areas experiencing an outbreak of the disease for the first time hence the focus of this study. This south western Kenya HAT foci is part of the Busoga HAT focus that combines both the western Kenya and eastern Uganda foci, covering an area of 3889Km^2^ and traversing Busia, Bungoma and Teso counties in Kenya. The 1990 epidemic outbreak affected both countries in the Busoga focus [22]. The ecosystem comprises hills, valleys and rivers draining into Lake Victoria harbours and is a habitat for *Glossina pallidipes* and *Glossina fuscipes* which are important vectors for animal and human trypanosomiasis, respectively [9]. The vegetation in the uncultivated land in the area is mainly composed of savannah grassland interspersed with *Lantana camara* bush and *Digithonia spp*, which form good habitats for tsetse flies. Thick forests and swamps are found along the rivers and streams, which form suitable habitats for *Glossina fuscipes*, a riverine tsetse species that is the main one infesting the areas [23]. The area has a population of about 6 million people, and is served by 200 health facilities [24]. The area also hosts the Alupe Health facility, the only referral hospital for sleeping sickness in Kenya established in 1970. Thirty eight of the households are female-headed while the mean household size is 4.6. The population age-sex structure is wide based with more persons in the younger age groups than in the older groups for both sexes [24]. A majority (60%) of the population fall below the official poverty line [13]

### Study design and selection of study participants

A retrospective cross-sectional study that applied mixed methods approach was used. To limit recall bias due to arise in any retrospective study, triangulation of data collection methods was used. Former HAT patients from the year 1990–2002 in the 13 villages that recorded high HAT numbers were the main respondents for this study.

Data were obtained through both quantitative and qualitative methods. Quantitative methods included review of hospital records from 1990–2002 to identify HAT patients and their villages, the stage of the disease at the time of diagnosis, and duration the patients spent in hospital. To identify past HAT patients, the study adopted the purposeful sampling strategy where the existing KETRI-Alupe HAT database formed the sampling frame. The databases were compiled according to districts, locations, sub-locations and villages, and dated as far back as the 1950s. However, for the purpose of this study, only patients from 1990 to 2002 who resided in the 13 villages that experienced the disease for the first time and recorded high HAT numbers were considered. During this period of study, there were 208 HAT patients in the database. Due to difficulties of tracing some of them as a result of migration and natural attrition, only 152 HAT patients or their guardians were traced with the help of the village chiefs and interviewed using a structured questionnaire. Five research assistants who were taken through a one-day training on the use of the tool helped administer the questionnaires. The quantitative data collected through this method included, socio-demographic and economic data, HAT treatment seeking behaviour and effects of HAT on the household including coping strategies.

Qualitative methods included key informant interviews, case narratives and focus group discussions (FGDs). Eleven key informant interviews were held with opinion leaders from key affected villages and health personnel who had attended to the HAT patients to understand the disease in the context of the study area. To be recruited as a key informant, one had to have lived in the study area during the 1990 HAT epidemic and been in a key position in leadership or a health facility. Participants for the case narratives were identified with the help of the health staff and village chiefs. Twelve case narratives were conducted with former HAT patients, who had various experiences that best illustrated the socio-economic impacts of HAT, to enable a richly detailed exploration of individual’s own perceptions and accounts of their experiences with HAT. These were identified and recruited during the administration of the structured questionnaire. In addition, 15 FGDs (5 male only, 5 female only and 5 mixed groups) were held in the study area. These consisted of between 6–12 participants who were identified and recruited on the basis of coming from the same village, having suffered from HAT or were guardians/care-takers of former HAT patients and their immediate neigbours. The FGDs were held to re-visit emerging issues especially with regard to the socio-economic effects of HAT and the coping strategies employed by HAT affected households. In setting up these groups, attention was paid to homogeneity of the participants to give ample room for free discussions. However, in some areas where there were few HAT patients, mixed FGDs were conducted. The discussions focused on what these people knew about HAT and its effects on the HAT patients and their households. A checklist was used to guide these discussions. The first author played the role of a moderator/interviewer and a trained research assistant took detailed notes during the FDGs, key informant interviews and the case narratives. These were also tape-recorded with the consent of the participants.

### Field work and data analysis

Field data were collected for 3 months, beginning January to March, 2004. We first approached the participants in person, explained the objectives of the study to them, and fixed an appointment with them for an interview/ group discussion based on their availability and convenience. The interviews were conducted in the homes of the HAT patients or a neutral quiet venue approved by the participants. All interviews lasted between 30 to 120 minutes.

To protect the informants from harm in terms of the nature of interviews, we took informed consent, assured them about anonymity, and told them that they were free to withdraw at any time or refuse to answer any specific question. The interviews were conducted in either the national language, Kiswahili, or the local languages.

All the recordings of the interviews were transcribed and translated into English. The hand written notes were used to fill in any gaps in the recording to complete the transcripts. Thematic analysis was used to analyse the qualitative data. Thorough familiarity with the responses was gained by reading and re-reading all of the transcripts. This helped to code and categorise the data according to emerging themes. Quantitative data were coded and analysed descriptively using the statistical package for social science.

## Results

### Socio-demographics

**Fig 1:**
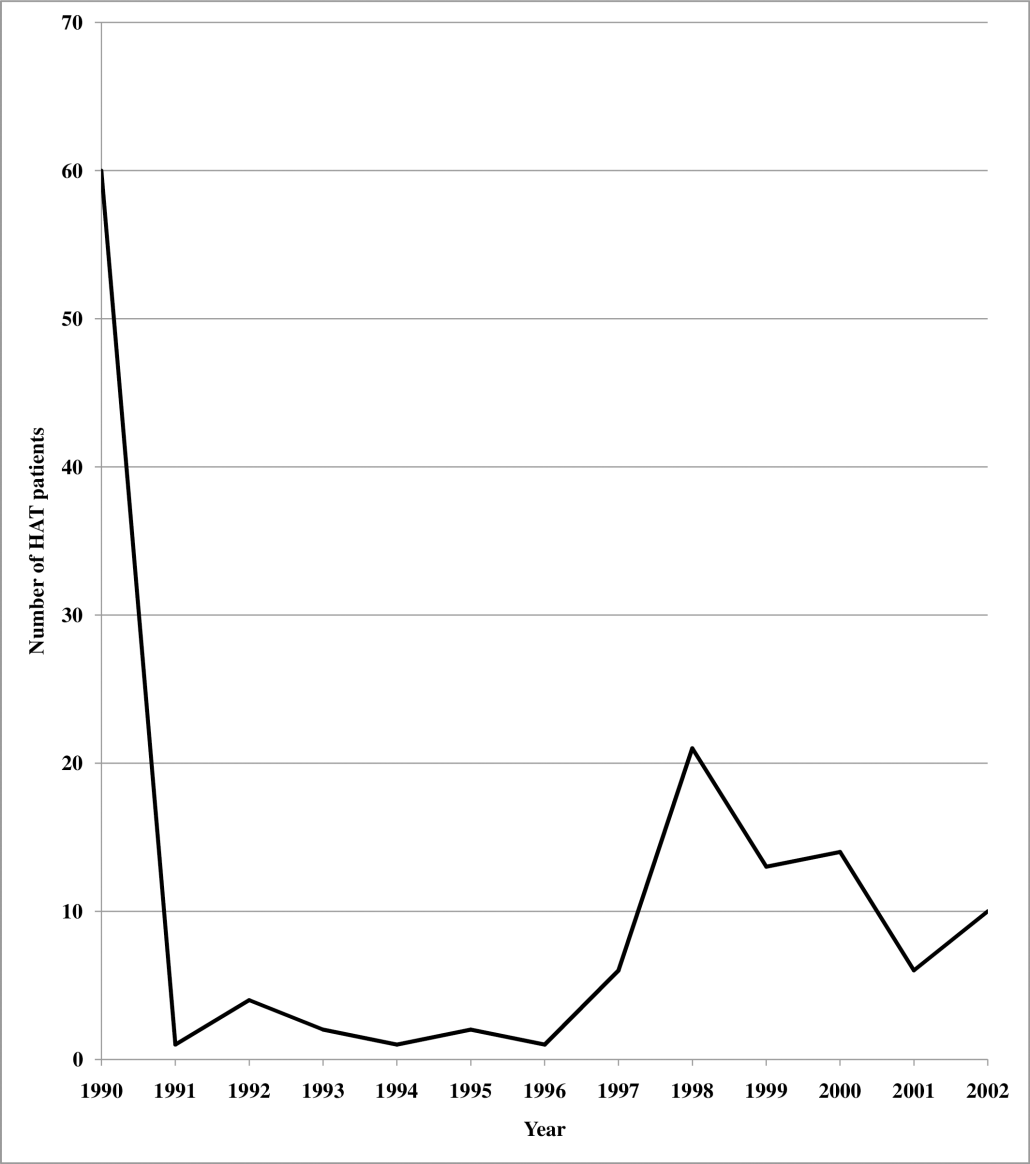
Trends in HAT patients in the south western Kenya HAT foci (1990–2002).

One hundred and fifty-two former patients who were recorded as HAT cases between1990-2002, (Fig 1) were interviewed to document the socio-economic impacts of HAT and the coping strategies of their households.

Table 1 presents a summary of the socio-demographics of the respondents. At the time of interviews, 88.8% (135/152) of former HAT patients or their guardians had only primary school or no formal education. A majority (77%) were married while 10.5% were widowed. Sixty three percent of the respondents were from monogamous households while 30% were from polygamous households. Up to 82% of the respondents were primarily farmers, and 76.9% of the respondents earned less than Kenya shillings 4000 (52 US dollars) per month (US 1 was an average of KES 77 during the period of data collection.

**Table 1:** Socio-demographics of HAT patients in the south western Kenya HAT foci (1990–2002) (n = 152).

Variable	Respondents	
	Frequency	Percentage
**Level of education**		
None	52	34.2
Primary	83	54.6
Secondary	14	9.2
Diploma	3	0.7
**Marital status**		
Single	16	10.5
Married	117	77
Separated/Divorced	3	2
Widowed	16	10.5
**No of wives in the home**		
1 Wife	96	63.1
2 Wives	25	16.4
>2 Wives	22	14.5
None	9	6
**Occupation**		
Farming	124	82.7
Business	6	4.0
Employed	10	6.6
Other	12	6.7
*Monthly income (KES)		
0–3999	117	76.9
4000–7999	24	15.8
8000–11999	10	6.6
12000 and above	1	0.7

* KES 77 is equivalent to US$ 1.00

Through a questionnaire survey, we determined the duration of illness before HAT diagnosis was made and treatment started. Only 15% of the patients reported a diagnosis of HAT following onset of clinical signs, with the majority (57.6%) starting HAT treatment after two months (Table 2).

**Table 2:** Health seeking behaviour in relation to HAT in south western Kenya HAT foci.

Variable	Months	Total (%)			Percentage
		Male	Female	Total	
***Duration before correct diagnosis and treatment (n = 106)***	<1	10	6	16	15.1
	1–2	16	13	29	27.3
	2–4	13	9	22	20.8
	>4	20	19	39	36.8
	**Stage**				
***Stage of disease at time of diagnosis (n = 152)***	Early	19 (12.5%)	21 (13.8%)	40	26.3
	Late	58 (38.2%)	54 (35.5%)	112	73.7
	**Amount (KES)**	**Total**			
***Amount spent (n = 106)***	<4000	74			69.8
	4001–8000	19			17.9
	8001–1199	6			5.7
	>12000	7			6.6

Additionally, 73.7% of the HAT patients were diagnosed at a late stage. Almost an equal number of male (12.5%) and female (13.8%) HAT patients were diagnosed with the early stage while slightly more males (38.2%) than females (35.5%) were diagnosed with the late stage of HAT, having sought treatment using various options (health facility, over-the-counter, traditional medicine, diviners and seeking prayers). There is no statistically significant association between sex and stage of diagnosis (Chi square = 0.2165, p = < .05).

### Socio-economic impacts of HAT

The main symptoms of the disease as mentioned by the respondents include sleep (69%), fatigue (60%), feeling cold (40%), loss of appetite (28%), body swellings (6%) and miscarriage in pregnant women (1%). However, in the focus group discussions, mental disturbance, weight loss, itching, rashes, joint pains, headache, stiff neck, nausea, partial blindness, stomach-ache and paleness of skin colour were mentioned. There was consensus among FGD participants on the similarities of HAT and AIDS. They were thus in agreement with the following statement from one of them whose son suffered from HAT:

People at first thought the disease was AIDS. We only came to discover it is not AIDS because my son as young as he was had the same signs as those of AIDS. There was no possibility of him being infected with HIV since we, his parents are not victims at all. **(Male FGD, Teso)**

More than a quarter of the respondents (30%) reported that the community members thought that their illness was caused by witchcraft, 21.2% of them related it to the tsetse fly, and 34.5% cited HIV/AIDS while 14.3% mentioned other varied causes such as malaria and malnutrition among others. The treatment options and pathways sought by the respondents and the delays in diagnosis are already a subject of detailed discussion in an earlier paper [25] emanating from this study.

In seeking varied alternative modes of treatment for HAT based on the manifested signs and symptoms and perceived causation of the disease households spent various amounts with more than half (69.8%) utilizing up to KES 4000 (Table 1). Others utilized much more as demonstrated by what is expressed by one of the affected:

For my brother’s case we believed it was witchcraft due to the different and complicated symptoms so it consumed a lot of our money. For instance, the first diviner consumed 10,000 Kenya shillings since he really convinced us that somebody had bewitched my brother. He therefore tried his “things” but they didn’t do my brother any good. We switched to another diviner from Kakamega who also tried in vain. Soon we realized that on the diviners alone, we had used almost 25,000 Kenya shillings without any improvement on my brother’s condition. It was not until he was taken to Alupe that he was diagnosed and found positive for sleeping sickness.

(32-year old brother to an 18-year old HAT patient, Case Narrative, Bungoma)

However, one key informant, a nurse, in the HAT hospital clarified that most respondents incurred these medical expenses before proper diagnosis was made.

Usually after trying other means of treatment which do cost them a lot of money that’s when they come to the hospital, so they use a lot of money. Patients take about a month or more before they come to the sleeping sickness hospital. They only come when their condition has deteriorated and they are not able to walk. **(Female, 40yrs, Nurse, Teso)**

She also clarified that once proper HAT diagnosis had been made, treatment was carried out free of charge, but patients and their households still incurred other indirect expenses such as transport costs to visit the patient and loss of working time and income.

The health seeking behavior of the respondents and their families in trying to get treatment for their illness from various options increased financial demands on 93% (106) of the affected households. Delays in getting a correct diagnosis emanated from both the nature of care seeking by the patients and their families, and from the health system as illustrated in the following excerpts:

At first we believed it was malaria so we had to move from one health centre to another in vain. We tried as a family and contributed money to take him to Bungoma District hospital where he was diagnosed but was found to have typhoid associated with acute malaria so they switched to treating malaria and typhoid but the patient’s condition did not improve. Having failed medically, we now came to believe it was witchcraft so we tried different diviners but there was no change. We were almost losing hope until the patient was finally taken to Alupe (HAT referral hospital) where he was diagnosed and found to be suffering from sleeping sickness.**(25-year old son to a 52-year old HAT patient, Bungoma)**

Sometimes we would miss a positive patient because it was difficult to test a patient with sleeping sickness when the parasitemia was very low in the blood. The diagnosis entailed the use of the microscope to screen blood and this was at times tiresome and cumbersome hence it was not an ideal method. We would also use mice to confirm suspected HAT cases but this would take time. **(Male, 39 yrs, Medical Lab Technoloigst, Teso)**

Cross tabulation of duration taken before correct diagnosis and average amount of money used showed that those who took more than one month before diagnosis utilized over KES 3000 meaning that delays of more than one month led to increased expenses. The average amounts spent were incremental with the time one spent before correct diagnosis. However, after staying for over four months before correct diagnosis, the amount spent reduced (Table 3).

**Table 3:** Cross tabulation of duration before correct diagnosis and amount spent (n = 106).

Duration	Average Amount Spent (KES)	Number of Patients	Percentage
<1	1200	16	15
1–2	4600	29	27.3
2–4	5000	22	20.7
>4	3200	39	37

**Fig 2:**
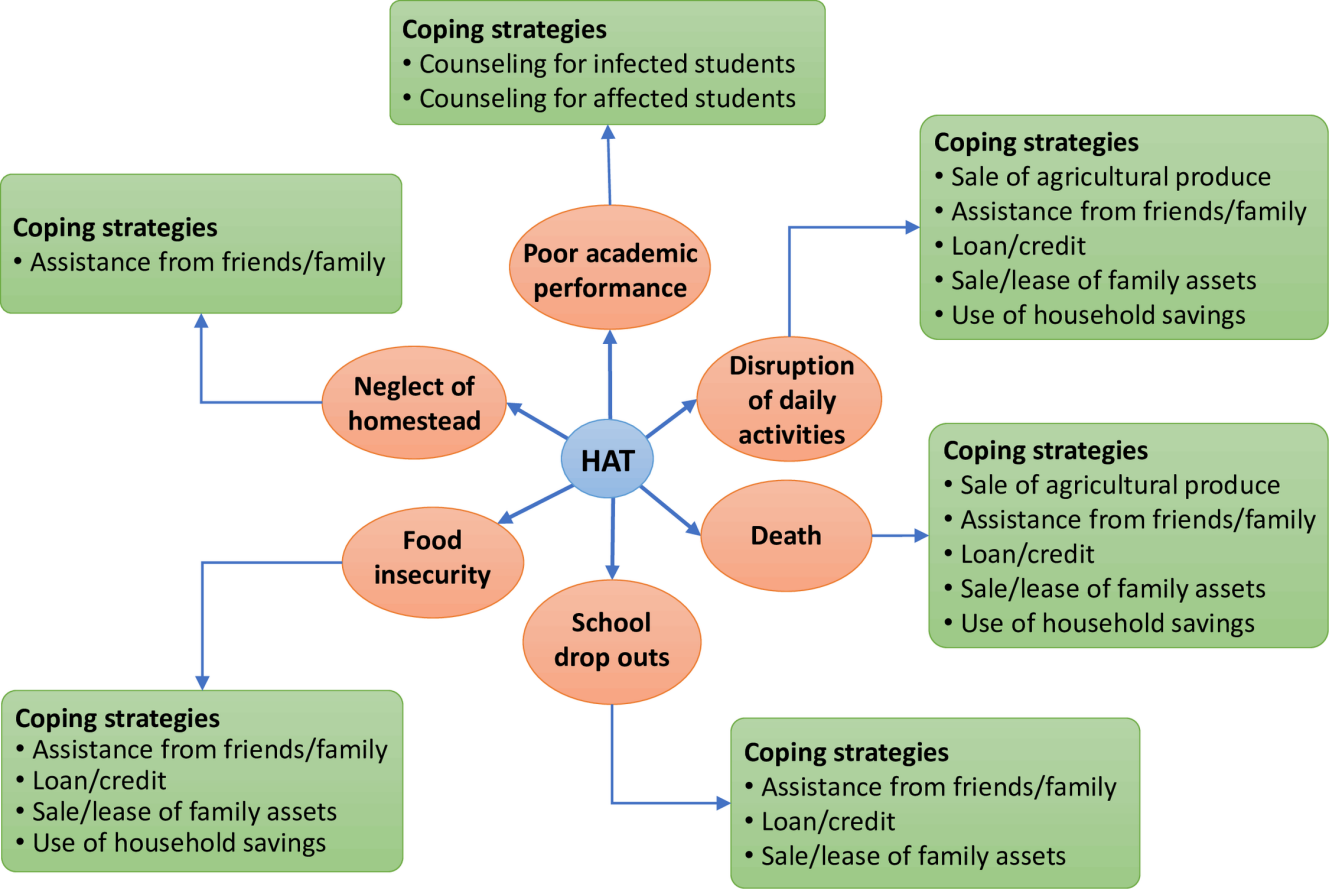
Socio-economic impacts of HAT and the coping strategies of households in the south western Kenya foci.

Almost (80%) of the HAT patients once correctly diagnosed, took an average of five weeks in hospital undergoing treatment. This combined with the process of seeking treatment led to various socio-economic impacts and various coping strategies that were utilized to deal with it as illustrated in Fig 2.

#### Disruption of daily activities

Disruption of daily activities was experienced, as many HAT patients could not continue with their daily routine activities including economic activities due to time spent in either seeking treatment or undergoing treatment in the health facilities. The impacts were more adverse if the HAT patient was either the breadwinner of the home or was in full time employment as illustrated by a health personnel:

*The treatment has not given patients any problems but those who were quite busy like teachers were affected a bit because of the time they spent in the ward at the expense of their jobs*. (***Female 43*, *Nurse*, *Teso***)

The activities of the immediate family members were also interrupted when labour was withdrawn from economic activities to provide care to the sick person. Discussants in the FGDs were in consensus that when a man was sick his wife or wives were forced to interrupt their daily activities to take care of him, while if a woman was sick, it was her co-wives (if she had any), her children or her sisters who would come in to take care of her and perform her domestic chores. In polygamous families, the burden of taking care of the sick husband mostly rested on the younger wife, who would usually be wrongly blamed for having infected the husband with HIV or having bewitched him, hence left to fend for herself, her children and her sick husband.

Hatred developed between me and my co-wife because she talked evil things about me saying that I had brought AIDS in the family with an intention of destroying her life- She left the whole burden of taking care of our husband on me yet i also had small children whom I needed to feed. **(Female, 32yrs,Teso)**

The Survey results indicated that about 10.5% of respondents were widowed. Widows or widowers with sick children also faced difficulties in terms of continuing to engage in their normal economic activities, as seen in the following excerpt:

In the process of raising money for my treatment, my widowed mother’s business collapsed since she used the business money for my treatment. She couldn’t continue with the normal farming activities. My siblings had to stay with my grandmother since my mother was busy looking for money and caring for me. **(Male, 19yrs, Case Narrative, Busia)**

Also depicted in the excerpt, is the role that maternal grandmothers played in helping take care of their grand children. In the case where a widow /widower were sick, extended family had to step in to.

#### Food insecurity

Food insecurity was cited as a concern as the productive members of the family spent a lot of their time and many days trying to seek for treatment. This meant that they either spent little time or none at all on their farms. Informants indicated that the disease struck at various times of the planting season. For some, it was during the time for preparing the land for planting, for others, it was during weeding time while for some others, it was during harvest time. The situation was more severe and conditions tougher on the women whose husbands had contracted the disease, because they lacked somebody to help them prepare the land for planting. Loss of oxen through sale or death to trypanosomiasis also contributed to food insecurity as elaborated in the following excerpt:

The farm remained untilled because people didn’t have the energy to work as most of those who had bullocks or oxen lost them to nagana (animal trypanosomosis) or sold them to meet financial expenses. We even sold four of our cattle to get finance to help us in seeking treatment. We even gave some to emuron (diviner) from whom we sought treatment. **(48-year old father to a former HAT patient, Case Narrative, Teso)**

At the same time of the 1990 HAT outbreak, it was reported that the disease also caused deaths in many livestock as exemplified by a key informant:

Looking at the economic activities of people living around here, they are mostly farmers. Their type of farming mostly depends on ox-ploughs and not tractors. Therefore as a result of trypanosomosis wiping out the cattle, the farmers were not in a position to till their land. Hence a lot of land was left fallow simply because tractors were expensive and at the same time they were few in the area hence it was not easy to get a tractor to till the land. Most people had no personal tractors hence it was difficult for them to plan their farming activities on time. **(Male, 51yrs, clinical Officer, Teso)**

Lack of oxen to plough meant that the size of land put under cultivation during that season was relatively small in comparison to other seasons This implied that the harvest for that season was also minimal and hence the hunger experienced by most of the HAT-affected households.

#### Neglect of homestead/loss of property

When most of the adults in the homestead were frequently either seeking treatment in hospitals or tending to the hospitalized, the homesteads were left unmanaged and prone to malicious damage or vandalization. One 18-year old former HAT patient reported that when he was admitted in hospital, his mother, a widow, had to stay with him for almost a month and left the home unattended leading to negative consequences as explained in the following statement:

Since my mother stayed with me in hospital for almost a month, there was no one to look after the home. When she went back home, the place was bushy, our chicken had been stolen and there was no food. **(Male, 18yrs, Case Narrative, Busia)**

Former male HAT patients confirmed this in an FGD and concurred with this statement, *“Since we were all in hospital*, *people stole our property*. *For example; bananas from the garden*, *chicken and household utensils among others*.*”*

#### Poor academic performance/school-drop-outs

Poor performance in school was reported to have been experience by some children either directly or indirectly affected by HAT. The children who suffered from HAT had to miss school several times due to ill health. Some of the HAT patients of school going age were forced by circumstances of ill health to miss school several times while others eventually dropped out of school. One 19-year old boy, during a case study interview, echoed this by saying:

“Sleeping sickness has interrupted my studies. My performance deteriorated and I had to stop schooling. I am quite sad because by now I should have been through with my secondary education”. **(Male, 19yrs, Case Narrative, Teso)**

Children whose parents or family members suffered from HAT also experienced schooling problems. Some of these problems were as a result of the families trying to cope with the financial constraints experienced as a result of seeking treatment. This led to diversion of school fees leading to missed school days or total drop out from schools. Apart from providing care for the sick and lack of school fess, lack of food also contributed to school absenteeism as shown in the following quote: *“Children couldn’t go to school on an empty stomach* (hungry) *since all the food that was there was sold to try to make the patient recover*.*”* A FGD with men who had to take care of their sick wives said as much: *“Some children stopped schooling to take care of either their sick mother or father or other children in the home*.*”* The increased responsibility on the school going children negatively affected their school attendance and by extension, probably their performance too.

#### Death

Death, an irreversible effect of HAT occurred in many homes. A 68 year old former nurse from the HAT hospital reported that during the period under review (1990 to 2002) more than 40 people died while undergoing treatment. She further claimed that many others may have died in the villages before being diagnosed with the disease. Death of HAT patients occurred in some homes and left a feeling of helplessness and economic loss among the surviving family members as explained by a key informant:

People couldn’t work because of the grief of their loss. This caused a lull in many economic activities. The deaths robbed families and the community valuable and productive members. **(Male, 45yrs, Village elder, Teso)**

Death also contributed to a myriad of other problems depending on the previous roles of the deceased in the home. A 41-year old widow narrated her ordeal thus: “*I was widowed after my husband died of sleeping sickness*. *Thereafter*, *life became very challenging for me in terms of acquiring food for the family and paying school fees for the children*. *This has lead me to live a poor life of begging*.*”* In addition, a nurse in one of the health facilities in one of the most affected villages reported that, *“when a husband dies*, *his wife may have challenges in fully supporting the feeding and schooling of her children hence increased school drop-outs and early marriages especially among the girls*. Widows also faced issues in relation to property inheritance as depicted in the following excerpt:

There was misunderstanding between me and my co-wives during my husband’s illness. When he died, my co-wives grabbed everything he had,money and property, and left me with nothing.So he left me in great poverty. **(Female, 39yrs, Busia, Kenya)**

Similarly, the death of an adult robbed the family of household labour as reported by a widow, *“when my husband died*, *I lacked somebody to slash and till for me the land in preparation for planting*.*”* Widowers also faced challenges in relation to food preparation and taking care of the children especially in monogamous families. Children, especially the elder ones, mostly girls, were forced by circumstances to undertake the domestic chores in the home, such as cooking, fetching water and firewood, This interfered with the children’s schooling leading to poor performance or school drop-outs.

Death also indirectly led to loss of time and income through expenses used for organizing burial ceremonies. It also meant the loss of valuable time for family members as they spent their productive time attending to funeral activities instead of engaging in more productive work This is expounded by a village elder in the following statement, ‘*Death increased the financial burden of the affected households and the community in meeting funeral and burial expenses especially given that the funeral ceremonies can last for more than two days*.*”* Households therefore adopted different coping strategies to deal with the burden of HAT.

### Coping strategies to deal with the socio-economic impacts of HAT

#### Sale of agricultural produce

Sale of agricultural produce was a key coping strategy for most households as some of the affected families had to sell some of their property or farm produce. Goats, cattle, sheep, chicken, maize, millet, groundnuts and beans were among the farm produce sold or given away to help in seeking treatment. A 42-year-old widow and former HAT patient from Teso district had this to say: *I sold all my food to meet hospital and drug expenses*, *and also to pay the diviner*. While a 30-year-old former HAT patient elaborated that: *My parents also had to part with their three cows in order to raise funds*. *I had also harvested some 40 kilograms of maize*, *which had to be sold to raise money for my treatment*. A wife to a former HAT patient also reiterated that: *We used a lot of money to get treatment*. *For instance*, *personally before counting what his brother gave*, *I sold one sack and three* *gorogoros* *(2kg tin) of finger millet and two sacks of maize*.

#### Sale/lease of family assets

Household property such as bicycles, mattresses and blankets were among the items sold while land was either sold or leased out to get money to cope with the financial constraints encountered while seeking treatment for HAT. Sale of family property occurred when households had to give away some of their household goods to pay for services rendered by diviners. Similarly, patients and their families sold some of their property to cater for the medical expenses incurred as illustrated by the following statements from two case studies:

*My mother was very worried about me and she sold part of our land and leased part of it to raise money for my treatment*. ***(Female*, *21yrs*, *Case Narrative*, *Busia)***

The emuron (traditional healer) took away my household items like pans, bed-sheets, blankets and our clothes. My family even had to slaughter a cow under the direction of one emuron and I was made to eat the raw meat yet I did not recover. The emurons just became rich at the expense of my sickness. **(Female, 33 yrs, Case Narrative, Teso)**

#### Seeking loans/credit

Other HAT patients and their households incurred debts, which became difficult to pay as shown in the following three excerpts from case study interviews:

*…we also borrowed KES 3000/- from a friend*. *The repayment of debts incurred during his hospitalization was challenging as the disease had rendered the family poor*. *To date I am still paying by doing manual jobs on her farm*. ***(*Female**, **39 yrs, Case Narrative, Busia)**

When I was sick, my father had to borrow money from his association which really gave him a hard time to repay. **(Male, 30yrs, Case Narrative, Bungoma)**

For my husband’s case it was initially mistaken for witchcraft because he was having a land dispute with his brother. We therefore took him to a diviner who required a lot of money, which we couldn’t raise so we had to come back home. We then decided to borrow money and take him to Nangina mission hospital where he was diagnosed with sleeping sickness. We had to go back home and borrow money for transport to Alupe (Sleeping Sickness referral Hospital). **(Female, 39yrs, Case Narrative, Busia)**

#### Use of own/household savings

An Assistant Chief whose father died of HAT had the following to say about how they coped with the increased expenses by using their savings:

Sleeping sickness affects one in a way that you find households spending a lot of their money including their savings to try save the patient. Eventually, you find that one has used all his/her resources to cater for medication, which eventually results in great poverty. **(Male, 38yrs, Key informant, Teso)**

#### Assistance from family/friends

HAT affected households were forced to turn to family and friends for assistance when things became too tight for them and their households. Some assistance was financial or in kind as illustrated by the following excerpts:

The biggest problem was that people in this community were greatly weakened and could not work. As a result, hunger set in. The wife who could work on the farm had to take care of you and as such could also not work on the farm. She could only go to the neighbours to be assisted with some little money or do some odd jobs in friends’ homes to get some money…. **(Male FGD, Busia)**

Non-financial support included prayers as exemplified by the statement of a village elder in Busia, who had fallen sick, *“My wife was very worried because she thought the worst would happen*. *Therefore she sought help from members of her church group who would gather from time to time at my home to support her and offer prayers for my quick recovery*.*”*

#### Counseling

This related more to children who were infected and affected by HAT and more so, those who experienced behaviour change or were affected mentally as demonstrated in the following transcription:

When my son got ill he got affected mentally and I have been taking him for some help from an Organisation in my village that tries to rehabilitate mentally challenged children but it has not helped to improve his performance in school. It is very frustrating and this has taken a lot of my time and money **(Male, 40yr, Case Narrative, Teso)**

## Discussion

HAT patients and their households experienced increased financial expenses in seeking for treatment and this led them to adapt various coping strategies which ended up increasing the burden on affected households. HAT patients and their households utilized varied options in seeking treatment sometimes making several visits to different health facilities before getting diagnosed for HAT. Given the challenge in diagnosis of HAT, patients utilized several options, including health facilities, diviners, traditional medicine and prayer to get relief from their illness. Frequent visits to the health facilities without getting a proper diagnosis led some patient and their households to utilize other options such as diviners and prayers. Misdiagnosis of HAT has been reported in other studies and is attributed to its similarity with other febrile illnesses common in HAT endemic areas, in their clinical manifestations [16]. Delays in appropriate diagnosis could also be attributed to low awareness of the disease among the affected community. This could lead to diverse etiological beliefs about the disease which in turn influence health seeking behavior making several people to use diviners among other non-HAT related options [12, 25, 26]. Health system delays in HAT diagnosis can also be as a result of low index of suspicion among health workers and inadequate diagnostic tools in the health facilities [16, 27]. These delays in diagnosis leading to use of multiple health seeking options contributed to increased financial and social constraints on the HAT patients and their households. As observed, the longer one took before correct diagnosis, the higher the financial expenses. However, those who took longer than four months before correct diagnosis registered a drop in the average amount spent as compared to those who took between one to four months. This could be as a result of households resigning to fate having sought all manner of treatment in vain, coupled with the exhaustion of funds to continue actively seeking health care. Similar observations have been documented in the case of chronic diseases where due to increased medical expenses in seeking health care coupled with the long duration of chronic illnesses, patients and their households especially those from low income countries record reduced allocation of expenditure on treatment for the same [28]. Delays observed in this study are similar to those of previous studies in Uganda and Kenya which recorded that slightly more than half of the patients had a delay of more than two months before being correctly diagnosed for HAT [25, 29]. The time between first awareness of symptoms to start of treatment is a matter of considerable public health concern because delayed treatment has implications not only on disease outcome but also on the economic and social aspects of patients and their household [16]. Most (73.4%) of the HAT patients in this study were diagnosed in the late stage and admitted for up to five weeks in hospital. Diagnosis with late stage *rhodesiense* HAT requires treatment using melarsoprol, which has serious side effects and relapses with the duration of hospitalization lasting up to 40 days [9].

Prolonged hospitalization with HAT reduces the capacity of the patients to participate in productive activities. This is potentiated by functional incapacities and increased dependency that also drains household resources [9]. Some HAT patients lost their jobs or businesses as a result of being away for long durations leading to lose of income. The quantity and quality of household labour was further affected as members of the affected households spent more time looking after the sick and less time in agricultural/economic pursuits. Just like in other studies on other diseases, [30, 31], the immediate family were identified as the main care provider of HAT patients in this study. This implied that households had to find ways of coping with decreased household labour and income and increased expenditure on health. The burden of care-giving fell heaviest on women, who lost productive time giving care. Similar findings were observed by [30] in studies on maternal mortality in western Kenya, who reported that according to the gender systems, women, who are the traditional caregivers, spent a considerable amount of time providing care related activities to their families. Invariably, when women are ill other productive members of the community, particularly female relatives, are drawn out to provide caretaker services [30]. Burden of disease measurements need to take such aspects into consideration given that the burden of disease is not felt equally across gender.

The costs of HAT drugs is met by the World Health Organization and hence the free treatment of HAT in the hospitals. Nevertheless, the results of this study indicated that patients often sought treatment from a range of other service providers including the HAT hospitals before getting a proper diagnosis thus incurring huge costs in excess of KES 8,000 (US$104). A similar pattern was observed in a study on economic burden of tuberculosis in South Africa [32].These are very high expenses considering that about 60% of people living in the foci live below the poverty line [13]. Consequently, HAT continues to ravage people already living in abject poverty as also noted by [9].

The disease is considered to have a case fatality rate of close to 100% [10] hence several deaths were reported in the study area during the epidemic outbreak. Death sets off a multitude of shocks to household’s economic wellbeing, particularly in rural economies where the household is the main economic unit providing most of its own subsistence needs and where there are seldom any social protection measures in place [30]. Similarly, in this current study, the death of a family member due to HAT was observed to put a strain on the already impoverished households in different ways. Given that the main type of farming in the study area was subsistence farming that heavily depended on manual labor which was drawn from the household, death of any member in the productive age-group robbed the household of part of its labour force. This can lead to reduced resources for buying and purchasing food with negative implications on the household food security. Similar findings have been reported in studies on HIV/AIDS and maternal mortality in Malawi and Kenya [30, 31]. Widows tended to have a challenge with farming because they lacked the men to do the initial farm preparation of slashing and tilling, roles which are traditionally allocated to men in these communities [33]. Death in the household also caused reversal of roles which when not well handled, led to negative impacts. Widowers were forced by circumstances to undertake reproductive roles like cooking and caring for the children. However, given that they were not well versed in these areas, older children especially girls were forced to undertake these domestic chores hence affecting their performance in school or causing them to drop out of school and get married. Death of a parent has been reported to negatively affect household nutrition and labour allocation of households leading to decreased education and early marriages of girls [30, 31].

The death of a family member due to HAT was observed to put a strain on the already impoverished households as they were faced with the impending task of meeting the burial expenses to give their departed relatives a decent burial. This led to shifting of time, labour and resources from productive work to activities related to burial preparations which could take about a week. According to Luhya customs, it takes about three days for a child’s funeral and about a week for a funeral of a adult and during this whole period people will camp in the home of the deceased to sooth the bereaved [34]. This has negative implications on the household’s time, labour and finances as also reported by [30]

The burden of the health care related costs were further exacerbated significantly by the costs incurred by households in funeral expenses. The effects were more severe on households that lost a breadwinner and by extension, foregone income earning opportunities. Given that HAT mainly affects the productive age group it robs the family of the main support pillar posing a threat to survival of families and triggers a host of sociological, economic and psychological effects on the children [31].

HAT imposed high costs on individuals and households over time due to frequent treatment seeking and incapacitation of the sick. The high costs of illness associated with HAT, often went beyond most households’ monthly budget. The coping strategies adopted to mobilize substantial additional sums of money to meet these costs led to undesired negative consequences. The cost burdens of HAT can be extremely high for poor households, forcing coping strategies that reduce their asset portfolios and increase vulnerability to future shocks.

The socio-economic impacts of HAT as revealed in this study were similar to those of other debilitating illnesses described elsewhere in eastern and southern Africa [31, 35]. The loss of adult labour leads to a suite of changes in affected households’ use of land and other resources. Agricultural activities were often delayed, with negative effects on production, and land was often left fallow. A study on HIV in sub-Saharan Africa indicates that surviving household members may be under increased pressure to seek agricultural labour, paid in cash or kind or may pursue non-agricultural income generating activities that yield a quick return [36]. In south western Kenya, the HAT epidemic contributed to this problem, because adults who were absent from home for long periods to nurse sick relatives in hospital could not properly care for and guard their own fields and animals. Similar studies [31, 32] also reported increased socio-economic impact due to loss of productive labour time for tuberculosis and HIV/AIDs respectively.

The current study revealed that households sold their food reserves, got assistance from family and friends, depleted savings, got credit or sold family assets. Selling in distress meant that the returns were poor and also contributed to increased indebtedness. This was in line with the findings from a global study on the economic and social burden of Tuberculosis [32]. The families who were forced by the nature of treatment of HAT to leave their children in charge of homes while they were in hospital, lost important assets like land, livestock or household goods. The vulnerability of children left to become decision makers, responsible for the day-to- day running of the homes, exposes them to being taken advantage of by other adults. A study on HIV/AIDS in Kenya, report cases of children taken advantage of by adults who either grab or swindle them out of their property [37]. Critical agricultural resources were thus lost by the affected households, further deepening impoverishment. Reduced production, consumption and income as a result of prolonged illness put household members other than the person with HAT at risk of malnourishment since reserve food stocks were the first to be disposed of in order to meet both the direct and indirect costs of treatment. Women in particular were left with few means to secure their own and their families’ living than to burden their own extended families or seek alternative means of survival such as providing labour in other people’s farms for a fee.

The main economic activity of communities in the south western HAT foci is farming as attested to by 80.8% of the respondents. Furthermore, the farming system here is labour intensive and draws its labour from the households [38]. Problems therefore set in when the labour force required to work on the farms was either lacking due to weakness caused by illness, or was diverted to caring for the HAT patient. This implies a shortfall in the labour required to work on the farms thus leading to poor harvests or none at all. [31, 32] report that HIV and Tuberculosis, respectively, lead to shortages of able-bodied adults, particularly in the peak seasons of planting and harvesting, resulting in low agricultural yields. HAT morbidity and mortality therefore affect food security by reducing the household’s ability to produce and buy food, depleting assets, and reducing the insurance value of social networks as the household consistently calls in favours [15]. HAT morbidity affects agricultural productivity by reducing labour availability, and forcing households to reallocate labour from agriculture to patient care, while mortality permanently removes adult labour from the household leading to foregone opportunities. This combination of adult morbidity and mortality and the associated reallocation and withdrawal of labour eventually leads to food insecurity.

Furthermore, the available food stocks held in reserve are sold to meet the cost of seeking treatment. The results of the study also showed that family members of HAT patients were not only consuming fewer meals but sometimes went hungry since there was no food and some were often too young to work for an income. This was especially true of young children whose parents were either both sick or were away in hospital. Livestock were also sold to generate cash for patient care; while some died due to disease or poor management. Households which lost livestock also lost manure for their farms, milk for the family and “ambulatory” savings in addition to oxen for ploughing [39]. Given that most farmers in this study area depend on draught power to prepare their land for cultivation, absence of oxen leads to reduced size of land put under cultivation leading to relatively low production and hence food insecurity [39].

Food security hinges on the presence of household assets, which create a buffer between poor years of crop production, on the one hand, and consumption and exchange for needs, on the other. In times of need, assets such as livestock, land and even furniture can be readily converted into cash to buy food. Households often accumulate assets as an insurance strategy, but HAT forces them to dispose of their assets. Livestock, agricultural produce and household assets are sold, while land is leased out to help meet increased medical expenses. This robs households of their security and buffer during tough times like when crops fail. Impoverishment of households and their long-term vulnerability are also reported in South Africa in respect to tuberculosis [35]. There is need to find a way of incorporating variables, key in HAT patients’ coping mechanisms, in estimating the real burden of HAT. The inclusion of coping strategies and indirect costs in burden calculation help to provide a comprehensive assessment of actual burden of disease [32]

### Limitations

This was a retrospective study hence there would have been some recall bias however, the study made use of triangulation of methods to try and deal with this challenge. Similarly, sleeping sickness is not a frequent disease in the study area and most households were experiencing it for the first time ever, hence they were able to recall their experiences quite vividly. This study has leaned more on the qualitative aspects given that most studies on burden of HAT are quantitative in nature. Much as the findings of the study cannot be generalized to all HAT endemic areas, given that the focus of the study was on the *rhodesiense* form of HAT, the findings are based on real experiences of HAT patients in seeking treatment thus highlight key issues necessary to be considered when calculating burden of the disease. The data for this study was collected in 2004 for a PhD study. Over the years, the dynamics of HAT in Kenya have changed with only four new cases being reported from the period 2004 to 2016 [40]. However, given the neglect of NTDS at International and national levels, partly due to the limited information on their real burden, and specifically, limited information on the effects of coping strategies on the socio-economic burden of *rhodesiense* HAT, the findings from this study will be useful in highlighting the real burden of HAT and by implication, other NTDS. This is important in the face of the WHO’s 2030 road map for accelerating work to overcome the global impact of NTDs including HAT elimination [2]. The paper also provides an advance in knowledge about HAT as one of the NTDs and provides a basis on which future research can build upon.

### Conclusions

The findings of this study show that HAT resulted in exorbitant in-direct health care costs (ranging from US$60–170) for people already living on less than US$1 per day, this leads to households engaging in coping strategies which further impact negatively on the effects of the disease on households. Morbidity from HAT temporarily removes adult labour from the household hence affecting labour availability, causing shifting of household roles with some children forced to be absent from school, or to drop out of school temporarily or permanently to provide household labour. The socio-economic effects of HAT is exacerbated by coping strategies with negative consequences on households, that erode their asset bases and makes them more vulnerable to any other future shocks. It also implies that the burden of HAT goes beyond morbidity and mortality to include other indirect costs that if left out would lead to misinterpretation of the actual burden of the disease and also condemning the families to further poverty. HAT is a disease that has the potential of negating the progress made in the achieving of the SDGs and elimination of HAT and therefore concerted effort should be made to enhance the capacity of health care facilities including provision of appropriate diagnostics to ensure prompt treatment at onset of disease. The community also needs to be empowered to help build and strengthen their livelihood base and assets in order to be resilient in the face of shocks such as those posed by HAT. Continuous sensitization and awareness creation about HAT at the community, national and international level is critical in making progress towards the goal of HAT elimination by 2020.

## Supporting information

S1 Quantitative Data (XLSX)

S1 Case Study Guide (DOCX)

S1 Key Informant Interview Guide (DOCX)

S1 Focus Group Discussion Guide (DOCX)

S1 Ethical Approval Letter (DOCX)
