# Supplementary material for: The socio-economic burden of human African trypanosomiasis and the coping strategies of households in the South Western Kenya foci
Source: PLoS Negl Trop Dis. 2017 Oct 26;11(10):e0006002. doi: 10.1371/journal.pntd.0006002 (PMC5675461; doi:10.1371/journal.pntd.0006002)
Supplement: S1 Case Study Guide — (DOCX) [file pntd.0006002.s002.docx]

**Case Narrative Guide**

**Case study number ______________**

1. **Biographical data**

- Age of respondent _____________
- Sex _______________
- Marital status ____________________
- Religious affiliation ________________________
- Level of education _______________________
- Relationship to household head ____________________

1. **Knowledge about sleeping sickness**

- What causes sleeping sickness?
- How does one get sleeping sickness?
- Where did you get information about sleeping sickness?
- Did you know about the disease before or after being infected by it?

1. **Narrative**

- Story about how the patient got sleeping sickness and experience he/she went through.

1. **Treatment sought/coping mechanisms developed**

- Where did you seek treatment?
- How long did it take before the patient was correctly diagnosed with HAT?
- Was the treatment successful?
- What problems did you encounter when seeking treatment?
- What problems did you face after treatment?

1. **Effects of the disease on the patient and family**

- How did the disease affect you?
- How did the disease affect your family?
- How many hours were spent looking after the patient?
- How much money did you spend on treating the disease?

1. **Advice to the community about sleeping sickness**

- What have you learnt from your experience with the disease?
- What advice would you give a sleeping sickness patient?
- What advice would you give the community?

**THANK YOU**
