# Supplementary material for: The socio-economic burden of human African trypanosomiasis and the coping strategies of households in the South Western Kenya foci
Source: PLoS Negl Trop Dis. 2017 Oct 26;11(10):e0006002. doi: 10.1371/journal.pntd.0006002 (PMC5675461; doi:10.1371/journal.pntd.0006002)
Supplement: S1 Key Informant Interview Guide — (DOCX) [file pntd.0006002.s003.docx]

### Key Informant Interview Guide

1. Knowledge about Sleeping Sickness

- Local names for Sleeping sickness?
- What causes it?
- Who do you consider to be most vulnerable to the disease?
- How do you recognise sleeping sickness?
- Give a background of the disease over the years.
- What is the current status of sleeping sickness?
- Is it increasing or decreasing?

1. Attitude towards sleeping sickness patients

- Do you know of any victims of sleeping sickness?
- How does the community treat them?
- Do they recover after treatment?

1. Consequences of the disease on the individual, household and community.

- How does the disease affect a person?
- What are the implications of sleeping sickness on the family?
- What are the effects of the disease on the community?
- What can be done by the community to help sleeping sickness patients?

1. Coping strategies employed by individuals, households and community.

- How do victims of sleeping sickness cope with the disease?
- How is the disease treated?
- Is it expensive to treat?
- What is being done to control the disease?

THANK YOU
