# Supplementary material for: The socio-economic burden of human African trypanosomiasis and the coping strategies of households in the South Western Kenya foci
Source: PLoS Negl Trop Dis. 2017 Oct 26;11(10):e0006002. doi: 10.1371/journal.pntd.0006002 (PMC5675461; doi:10.1371/journal.pntd.0006002)
Supplement: S1 Focus Group Discussion Guide — (DOCX) [file pntd.0006002.s004.docx]

**Focus Group Discussion Guide**

1. **Definition of Sleeping Sickness**

- Local names for sleeping sickness
- Knowledge about the causes of the disease

1. **Symptoms of HAT**

- Identification of sleeping sickness.

1. **Vulnerability**

- Who is affected by sleeping sickness?
- Why are they vulnerable to the disease?
- How often do people get sleeping sickness?

1. **Treatment/Prevention/Control**

- How is it treated?
- How can it be prevented?
- What are you doing to prevent the disease?
- What is the community doing to prevent/control the disease?
- What can be done to improve on the control measures?

1. **Community reactions to sleeping sickness patients**

- What are your reactions to sleeping sickness patients?
- What are the community reactions to sleeping sickness patients

1. **Community practices as concerns sleeping sickness**.
2. **Social consequences of Sleeping sickness**.

- What are the effects of sleeping sickness on the individual, family, community

**THANK YOU**
