# Supplementary figures and images for: The socio-economic burden of human African trypanosomiasis and the coping strategies of households in the South Western Kenya foci
Source: PLoS Negl Trop Dis. 2017 Oct 26;11(10):e0006002. doi: 10.1371/journal.pntd.0006002 (PMC5675461; doi:10.1371/journal.pntd.0006002)

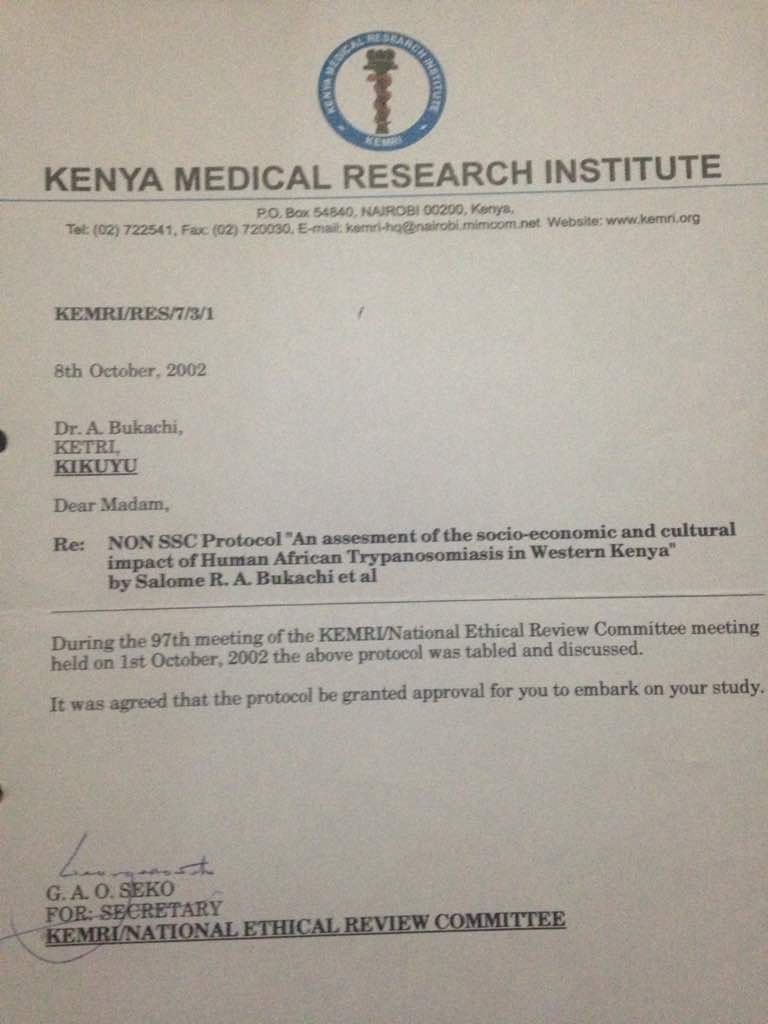

Supplement: S1 Ethical Approval Letter — (DOCX) [file pntd.0006002.s005.docx]
